# The suicidal survivor: Considerations for treatment in psychodynamic psychotherapy

**DOI:** 10.1192/j.eurpsy.2021.2195

**Published:** 2021-08-13

**Authors:** A. Maia Andrade De Souza, G. Lessa De Souza Maia, L. Helena Freitas Machado

**Affiliations:** 1 Medicine, Centro Universitário Tiradentes, Maceió, Brazil; 2 Psychiatry, Universidade Federal do Rio Grande do Sul, Porto Alegre, Brazil

**Keywords:** Suicide, Survivor, psychotherapy, trauma

## Abstract

**Introduction:**

Suicide is a phenomenon that is increasing in prevalence. Exposure to suicide by a loved one can be experienced as a traumatic event, capable of precipitating or aggravating preexisting psychiatric conditions. As much as we are clinically aware of gravity situation experienced by suicide survivors, there is a marked lack of studies on psychotherapeutic interventions in this population group.

**Objectives:**

The present work aims to review the literature on the psychodynamic treatment of suicide survivors, considering their theoretical and technical aspects.

**Methods:**

Narrative review of psychiatric and psychoanalytic literature.

**Results:**

The initial reaction described on becoming aware of the suicide of someone close to you is of disbelief, shock and helplessness. This is followed by ambivalent feelings of hate and guilt, shame and hopelessness. Sometimes, a chronic depressive state expressed by the survivor’s guilt can emerge. The mourning work will initially encounter resistance to face the loss of the object, through mechanisms such as denial, repression and psychotic fantasies. The lost suicide has a traumatic impact, modifying relational patterns and it is commonly associated with important isolation. The survivor will be able to transfer via fear the death of the therapist and even fantasize that he will also kill himself.

**Conclusions:**

Psychodynamic psychotherapy with suicide survivors finds theoretical and practical foundations in the literature, mainly through discussions of reports clinical and theoretical reviews on the topic. Through transfer and therapeutic alliance, new patterns of object relation can be sketched, in a context of mourning so often complicated by shutdown pressures and loneliness.

**Disclosure:**

No significant relationships.

